# Clinical Application of Machine Learning Models for Early-Stage Chronic Kidney Disease Detection

**DOI:** 10.3390/diagnostics15202610

**Published:** 2025-10-16

**Authors:** Hasnain Iftikhar, Atef F. Hashem, Moiz Qureshi, Paulo Canas Rodrigues

**Affiliations:** 1 Department of Statistics, University of Peshawar, Peshawar 25120, Pakistan; 2 Department of Statistics, Quaid-i-Azam University, Islamabad 45320, Pakistan; moiz@stat.qau.edu.pk; 3 Department of Mathematics and Statistics, College of Science, Imam Mohammad Ibn Saud Islamic University (IMSIU), Riyadh 11432, Saudi Arabia; affaragalla@imamu.edu.sa; 4 Department of Statistics, University of Sindh, Hyderabad 76080, Pakistan; 5 Department of Statistics, Federal University of Bahia, Salvador 40170-110, Brazil; paulo.canas@ufba.br

**Keywords:** chronic kidney disease, machine learning, clinical decision support, ensemble learning, early diagnosis, health informatics

## Abstract

**Background/Objectives:** Chronic kidney disease (CKD) is a progressive condition that affects the body’s ability to remove waste and regulate fluid and electrolytes. Early detection is crucial for delaying disease progression and initiating timely interventions. Machine learning (ML) techniques have emerged as powerful tools for automating disease diagnosis and prognosis. This study aims to evaluate the predictive performance of individual and ensemble ML algorithms for the early classification of CKD. **Methods:** A clinically annotated dataset was utilized to categorize patients into CKD and non-CKD groups. The models investigated included Logistic Regression, Linear Discriminant Analysis (LDA), Quadratic Discriminant Analysis (QDA), Ridge Classifier, Naïve Bayes, K-Nearest Neighbors (KNN), Decision Tree (DT), Random Forest (RF), Support Vector Machine (SVM), and Ensemble learning strategies. A systematic preprocessing pipeline was implemented, and model performance was assessed using accuracy, precision, recall, F1 score, and area under the receiver operating characteristic curve (AUC). **Results:** The empirical findings reveal that ML-based classifiers achieved high predictive accuracy in CKD detection. Ensemble learning methods outperformed individual models in terms of robustness and generalization, indicating their potential in clinical decision-making contexts. **Conclusions:** The study demonstrates the efficacy of ML-based frameworks for early CKD prediction, offering a scalable, interpretable, and accurate clinical decision support approach. The proposed methodology supports timely diagnosis and can assist healthcare professionals in improving patient outcomes.

## 1. Introduction

Kidney function gradually declines in CKD, an irreversible and progressive disorder that affects the kidneys’ capacity to filter blood and efficiently remove waste products [1]. Significant systemic health problems result from the accumulation of metabolic waste in the body when renal function deteriorates. Age and gender are two demographic factors that affect the disease, which is frequently linked to underlying problems including diabetes mellitus, hypertension, and cardiovascular disorders [2,3,4]. It is challenging to diagnose early, as symptoms typically appear later and include back and stomach pain, fever, rashes, and vomiting [5,6]. End-stage renal disease (ESRD) is indicated by values below 15 on the estimated glomerular filtration rate (e-GFR), which is one measure of clinical progression [7,8]. In these situations, kidney transplantation is still the only practical long-term therapy option when dialysis is not available [9].

Due to its asymptomatic nature, CKD is usually underdiagnosed in its early stages, despite its global prevalence [10]. While death rates are largely steady, the rising hospital admission rate—reported at 6.23% annually—underlines the increased healthcare burden associated with CKD [11]. Conventional diagnostic techniques for CKD are inadequate, emphasizing the need for computational algorithms that allow earlier and more accurate identification of high-risk patients [12,13]. In this context, ML and data-driven analytics are increasingly recognized as practical tools for developing clinical decision support systems (CDSSs) tailored to the needs of personalized medicine. ML enables the analysis of complex datasets derived from electronic health records (EHRs) and laboratory results, revealing hidden patterns and supporting predictive decision-making [14,15,16,17,18]. Through feature extraction and classification, these technologies enable clinicians to make individualized diagnoses and treatment decisions, even in data-rich yet insight-poor environments [19,20,21].

Recent research has shown that multiple machine learning methods may accurately predict CKD. Using six classifiers, such as Logistic Regression (LR), Random Forest (RF), Support Vector Machine (SVM), K-Nearest Neighbors (KNN), Naïve Bayes (NB), and Feedforward Neural Network (FF-NN), provided promising results [22]. RF obtained an accuracy of 99.75%. Similarly, the work in [23], developed a neural network model that predicted the start of chronic renal disease with 95% accuracy. Ref.  [24] employed SVM to achieve 93% accuracy, whereas [25] examined multiple classifiers on UCI datasets.

Further validation of the potential of ML-based CDSSs in early CKD identification has been provided by numerous additional contributions. The studies  [26,27] highlighted the importance of customized algorithms for subgroups by focusing on diabetic patients, a population at risk for CKD. In [28], a multiclass decision forest model with 99.1% accuracy was developed using a UCI dataset with 14 features. Ref. [29] employed a two-stage SVM method, achieving an accuracy of 98.5%. Other models, such as ANN in [30], Gradient Boosting in [31], and KNN in [32], have also demonstrated excellent accuracy on various datasets. Recent developments include hybrid techniques that employ optimization algorithms, such as ACO and Relief, as well as the use of deep learning [33]. Ref. [34], as well as comparative analyses on various CKD datasets have used up to nine ML classifiers [35,36]. Therefore, all of these results highlight how machine learning is increasingly changing traditional nephrology through personalized decision-making and predictive analytics.

Thus, by empirically comparing individual and ensemble machine learning models, the current study contributes to the evolving field of CKD decision assistance. We evaluate nine classifiers (RF, SVM, KNN, and Logistic Regression) and ensemble methods (Voting and Stacking) for diagnostic performance using several assessment metrics. To enhance computational efficiency and clinical applicability, we further investigate the function of feature selection and confirm our results using 5-fold and 10-fold cross-validation procedures. With the use of ML techniques, the main goal of this research is to create a reliable and understandable clinical decision support system (CDSS) for the early detection of CKD. The study highlights the use of predictive modeling to guide prompt, customized treatment interventions, aligning with the objectives of personalized medicine. Eight different ML classifiers were used to assess the prediction power of various algorithmic paradigms thoroughly. These include both linear and nonlinear models capable of capturing complex relationships within patient data.

To enhance the generalizability and reduce overfitting, both 5 and 10-fold CV techniques were adopted. These resampling methods provide a robust framework for estimating the models’ performance and ensuring their stability across different data partitions. The evaluation focused on key performance metrics such as accuracy, sensitivity, specificity, and area under the ROC curve (AUC), thereby offering a detailed comparison of each model’s diagnostic utility. This rigorous and systematic approach not only validates the predictive strength of individual and ensemble classifiers but also contributes to the broader objective of integrating ML-driven CDSS into nephrology for personalized risk stratification and early intervention.

The rest of the work is structured as follows: Section 2 explains the dataset and preprocessing steps, feature selection techniques, and the machine learning classifiers employed. It also outlines the implementation details, cross-validation procedures, and evaluation metrics used for assessing the model. Section 3 reports the experimental results, compares classifier performance with and without the feature selection process, and Section 4 discusses their clinical implications and outlines future research directions for enhancing CKD prediction using machine learning. Finally, Section 5 highlights the key contributions and emphasizes the importance of the findings.

## 2. Materials and Methods

### 2.1. Dataset Overview

This study utilizes a publicly accessible datasets that includes demographic and clinical data from individuals with and without chronic kidney disease (CKD). The dataset was initially gathered from the Burner Medical Complex (BMC), a medical facility situated in a rural area of Khyber Pakhtunkhwa in northern Pakistan. It was obtained using ResearchGate. With 258 (67.5%) CKD and 124 (32.5%) non-CKD cases, the dataset showed a rather unbalanced distribution. Stratified sampling was used to maintain the original class distribution across all subsets after the dataset was divided into training (70%), and testing (30%) sets in order to ensure a robust model evaluation. To ensure that observations are independent, each row represents a distinct patient. The dataset contains 21 therapeutically significant characteristics, including hemoglobin, albumin, sugar, pH, specific gravity, urine clarity, age, gender, and different blood cell counts. Demographic characteristics, such as age and gender, were added as predictors in addition to blood and urine measurements. Prior research has demonstrated that demographic factors can impact the occurrence and course of chronic kidney disease, even if these biomarkers are not diagnostic [37,38]. By incorporating these characteristics, the model can take patient variability into consideration. The significant variations across groups are highlighted in Table 1, which presents a full summary of these features stratified by CKD status. Nevertheless, the dataset has intrinsic limitations. Given that all patient information was gathered from a single, geographically focused healthcare facility, the study population may not accurately represent the broader clinical, environmental, and demographic diversity observed in other parts of Pakistan or elsewhere. In other places, factors such as genetic variability, environmental exposures, healthcare availability, and regional food practices may have a significant impact on how diseases emerge and how well models perform. Additionally, the dataset is missing crucial sociodemographic parameters that could enhance model generalizability and offer deeper insights into CKD risk factors, such as occupation, income, education level, and ethnicity. Therefore, it is important to exercise caution when extrapolating the results and prediction models from this dataset to other populations. Future studies should focus on validating these models using multi-center, demographically diverse datasets to enhance their applicability and external validity.

All analyses were conducted in the R programming language (version 4.3.2) using several specialized software packages: randomForest (version 4.7-1.1) [39] for Random Forest, e1071 (version 1.7-14) [40] for Support Vector Machines (SVM), xgboost (version 1.7.7.1) for Gradient Boosting, ggplot2 (version 3.5.1) [41] for data visualization, caret (version 6.0-94) [42] for training and evaluating machine learning models, and dplyr (version 1.1.4) [43] for data manipulation.

### 2.2. ML Methods

This study frames CKD detection as a binary classification problem using supervised learning algorithms to predict disease status. Let $X=[x_{1},x_{2},\ldots ,x_{n}]$ represent the predictor variables, and Y∈{0,1} the binary outcome, where Y=1 indicates CKD and Y=0 indicates no CKD. A variety of machine learning models—LR, LDA, QDA, DT, RF, SVM, NB, KNN, and Regression Trees—were employed to identify discriminative patterns for CKD diagnosis.

To evaluate model performance and mitigate overfitting, both the 5- and 10-fold stratified CV were implemented. Stratified CV ensures that class proportions are preserved in each fold. Since each patient appears only once in the dataset, data independence was maintained. Feature selection techniques were also applied to enhance model performance and reduce computational complexity.

#### 2.2.1. Logistic Regression

Logistic Regression (LR) is a commonly used classification technique that estimates the probability of a binary response based on input variables. Unlike linear regression, which models continuous outcomes, LR models the log-odds of the outcome:

(1) $$\mathrm{logit}\left(P\right)=\log\left(\frac{P}{1-P}\right)=\beta_{0}+\beta_{1}X_{1}+\ldots +\beta_{n}X_{n}$$

where *P* is the probability of the positive class and $\beta_{i}$ are the model coefficients [44].

#### 2.2.2. Linear Discriminant Analysis

The goal of LDA is to identify the linear feature combination that best distinguishes between two or more classes. It assumes that class covariances are equal and that features are normally distributed. Based on the Bayes theorem, LDA models the conditional probability P(Y=k∣X) for every class *k*:

(2) $$P\left(Y=k|X=x\right)=\frac{\pi_{k}\cdot N\left(x|\mu_{k},\Sigma \right)}{\sum_{j}\pi_{j}\cdot N\left(x|\mu_{j},\Sigma \right)}$$

In which $\pi_{k}$ represents the prior probability and N represents the multivariate normal distribution.

#### 2.2.3. Quadratic Discriminant Analysis (QDA)

QDA is a generalization of LDA that allows each class to have its own (covariance) matrix. This flexibility results in a quadratic decision boundary, improving accuracy in datasets with heterogeneous class distributions [45].

#### 2.2.4. Decision Tree (DT)

DTs are hierarchical models that split data into branches based on feature values, aiming to maximize class purity (e.g., using Gini impurity or entropy) at each node. They are interpretable but prone to overfitting [46].

#### 2.2.5. Random Forest (RF)

Random Forest is an ensemble of Decision Trees trained on bootstrap samples of the dataset. The final prediction is decided by the majority vote of each tree for a class. Mathematically

(3) $$H\left(X\right)=\mathrm{mode}\{h_{1}\left(X\right),h_{2}\left(X\right),\ldots ,h_{T}\left(X\right)\}$$

where $h_{t}\left(X\right)$ is the prediction from the *t*th tree, and *T* is the total number of trees [47].

#### 2.2.6. Support Vector Machine (SVM)

Support Vector Machines (SVM) construct an optimal hyperplane that maximizes the margin between two classes. For non-linear data, kernel functions (e.g., RBF) are employed to map features into higher-dimensional spaces, thereby improving separation [48].

#### 2.2.7. Naïve Bayes (NB)

Based on the Bayes theorem, the Naïve Bayes classifier assumes feature independence given the class label. The posterior probability is calculated as

(4) $$P\left(A|B\right)=\frac{P(B|A)\cdot P(A)}{P(B)}$$

This assumption simplifies computation and is effective even with small datasets [49].

#### 2.2.8. K-Nearest Neighbors

KNN is a non-parametric classification technique that uses the majority vote among the *K* nearest training instances, usually determined by Euclidean distance, to provide a class label. A careful selection of *K* is necessary, as it has a significant impact on model performance.

#### 2.2.9. Stacking Ensemble Learning

An Ensemble learning method called Stacking combines the projections of several base classifiers using a meta learner that is trained to maximize the output by analyzing the predictions of the base models. The base models are trained on the original feature set, and their outputs serve as inputs to the meta-model, which produces the final prediction. In this study, we employ Multiple Linear Regression (MLR) and Probability Distribution (PD)-based stacking to aggregate model predictions and enhance overall performance [50,51,52].

Mathematically, $D={\left\{\left(x_{i},y_{i}\right)\right\}}_{i=1}^{n}$, the base learners generate

(5) $${\hat{y}}_{i}^{\left(j\right)}=M_{j}\left(x_{i}\right),\;j=1,2,\ldots ,k$$

These forecasts are then combined by the meta-learner *F* to yield the final result:

(6) $${\hat{y}}_{i}=F\left({\hat{y}}_{i}^{\left(1\right)},{\hat{y}}_{i}^{\left(2\right)},\ldots ,{\hat{y}}_{i}^{\left(k\right)}\right)$$

### 2.3. Feature Selection

In order to evaluate the effects of dimensionality reduction, a mix of correlation analysis, univariate statistical tests (such as chi-square and *t*-tests), and recursive feature elimination (RFE) with cross-validation was used to choose features. Key indicators that are clinically relevant to the course of chronic kidney disease (CKD) were identified through this procedure, including serum creatinine, blood urea, albumin, hemoglobin, and hypertension.

### 2.4. Validation of Classifier Performance

All classifier training and validation procedures were executed using Jupyter Notebook (version 7.0.8). To ensure robustness and minimize overfitting, the dataset was partitioned using both 5 and 10-fold stratified CV techniques. These approaches maintain class distribution across folds, guaranteeing reliable performance assessment. An evaluation matrix comprising standard classification metrics was developed to compare the predictive accuracy of each model.

### 2.5. Evaluation Metrics and Confusion Matrix

A confusion matrix, a 2×2 table that summarizes the classification results, was used to evaluate classifier performance. The four main components of the matrix are false positives (FP), false negatives (FN), true positives (TP), and true negatives (TN). Key evaluation metrics, explained below, are made easier to calculate using this data.

(7) $$\mathrm{Accuracy}=\frac{TP+TN}{TP+TN+FP+FN}\times 100,$$

(8) $$\mathrm{Sensitivity}=\frac{TP}{TP+FN},$$

(9) $$\mathrm{Specificity}=\frac{TN}{TN+FP}$$

(10) $$\mathrm{Precision}=\frac{TP}{TP+FP}\times 100,$$

(11) $$F1\text{-}\mathrm{Score}=2\times \frac{\mathrm{Precision}\times \mathrm{Sensitivity}}{\mathrm{Precision}+\mathrm{Sensitivity}}.$$

## 3. Results and Interpretations

Several ML models for predicting CKD are thoroughly evaluated in this section. Detailed comparisons across various metrics, including accuracy, sensitivity, specificity, precision, and F1 score, are presented in tables and figures. The model’s performance is evaluated through comprehensive cross-validation and visualizations.

### 3.1. Performance Comparison Without Feature Selection

Without feature selection, Table 2 presents a summary of the classification performance of a few chosen machine learning models using the 5- and 10-fold CV method. For each algorithm, the optimal hyperparameters are found in Table 3 using a grid search strategy combined with 5-fold and 10-fold cross-validation. The performance metrics of the models following tuning under both cross-validation procedures are shown in Table 2. All evaluation measures showed that RF and Support Vector Machine (SVM) performed better than the other classifiers. Interestingly, in 5-fold cross-validation, RF obtained a sensitivity and F1 score of 0.9331, indicating a robust capacity to identify CKD cases while balancing recall and precision [7]. SVM shows comparable performance, particularly in precision and F1 score. LR, LDA, and Ridge Classifiers also show competitive results. The consistent results between 5-fold and 10-fold strategies demonstrate the robustness and generalizability of these models, indicating minimal risk of overfitting. Nonetheless, further performance enhancements can be achieved through hyperparameter tuning or the incorporation of ensemble approaches [53,54].

### 3.2. Impact of Feature Selection

Table 4 evaluates the effect of feature selection on model performance. Most classifiers exhibit marginal improvements in sensitivity and F1 scores. For instance, QDA demonstrates a notable increase in sensitivity from 0.8452 to 0.9414, underlining the importance of reducing feature space for better generalization. Both Logistic Regression and Random Forest benefit slightly from feature selection, reinforcing its utility in simplifying model complexity without compromising accuracy [54,55].

### 3.3. Feature Importance Analysis

Due to their built-in feature selection, tree-based models (such as Random Forest and XGBoost) performed better after feature selection, even though models like Logistic Regression and SVM performed better initially. Table 5 provides an overview of the chosen features and the corresponding model-wise performance gains [28].

In addition, Table 5 presents the relative importance of features across different classifiers. In this study, the final feature set included all predictors that were both clinically relevant to chronic kidney disease (CKD) and statistically significant (p<0.05), rather than arbitrarily selecting a fixed number of top-ranked variables (e.g., top 3, 5, or 10). These selected features were provided as inputs to both the Stacking Ensemble meta-learner and all individual base learners. Regularized linear models (e.g., Logistic Regression, Ridge) adjusted their coefficients based on the penalization strength, while tree-based models (Random Forest and XGBoost) further refined feature contributions using their inherent importance measures. This evidence-driven approach ensured the inclusion of only informative and non-redundant predictors, thereby maintaining both model accuracy and interpretability.

Among the predictors, *pus_cells* emerged as the most influential feature, particularly in Random Forest and SVM, aligning with established medical evidence [56]. Other key predictors included *albumin* and *bacteria*, both of which were strongly associated with kidney dysfunction [57]. Conversely, features such as *age* [58] and *bpigment* [59] demonstrated comparatively low predictive power. Overall, these findings highlight the dominant role of urinalysis-related indicators in CKD prediction [60].

Specifically, feature importance rankings were computed for the tree-based models, and SHAP (SHapley Additive exPlanations) value analysis was applied to the Random Forest and SVM classifiers to quantify the contribution of individual features to specific predictions. SHAP values provide a unified, game-theoretic framework for interpreting model outputs, allowing for the decomposition of each prediction into additive feature contributions. This approach not only corroborates the global importance patterns observed in Table 5 but also provides local interpretability for individual patient cases. By making the reasoning process behind model predictions transparent, this analysis enhances clinician trust and supports potential clinical validation in real-world settings.

### 3.4. Performance of Ensemble Models

The performance comparison presented in Table 6 demonstrates the superior predictive capabilities of Ensemble learning methods over individual classifiers in the early detection of CKD. Among all evaluated models, the Stacking Ensemble consistently achieved the highest metrics, with an accuracy of 0.9053, a sensitivity of 0.9498, a precision of 0.9044, and an F1 score of 0.9265. These results highlight its robust ability to correctly identify CKD cases while maintaining a balanced trade-off between false positives and false negatives. The Voting Ensemble also delivered a competitive performance (accuracy = 0.8974, F1 score = 0.9180), exceeding many of the individual classifiers in sensitivity and F1 score. Among the standalone models, SVM emerged as the most effective, matching the Stacking model in sensitivity (0.9498) and achieving a strong F1 score of 0.9228. QDA and Ridge Classifier also showed commendable results, with F1 scores of 0.9184 and 0.9193, respectively. In contrast, the Decision Tree classifier underperformed, particularly in accuracy (0.8474) and F1 score (0.8771), which may be attributed to its tendency to overfit on smaller datasets. Additionally, The confusion matrix in Table 7 demonstrates that the Stacking Ensemble model accurately recognized 118 out of 142 non-CVD cases (specificity = 82.98%) and 228 out of 240 CVD patients (sensitivity = 94.98%). With an F1 score of 92.65%, accuracy of 90.53%, and precision of 90.44%, the model performs well overall, especially when it comes to identifying real CVD cases. This makes it a good fit for clinical decision support, where reducing missed diagnoses is crucial. Overall, these findings affirm that Ensemble learning—particularly stacking—enhances generalization by leveraging the complementary strengths of base models. This capability is especially valuable in clinical decision support systems where both diagnostic accuracy and reliability are critical. Such approaches align with recent studies advocating for hybrid and ensemble models in medical diagnostics [59,61], underscoring their practical utility in data-driven, patient-centered healthcare.

### 3.5. Visual Interpretation

**Figure 1:**
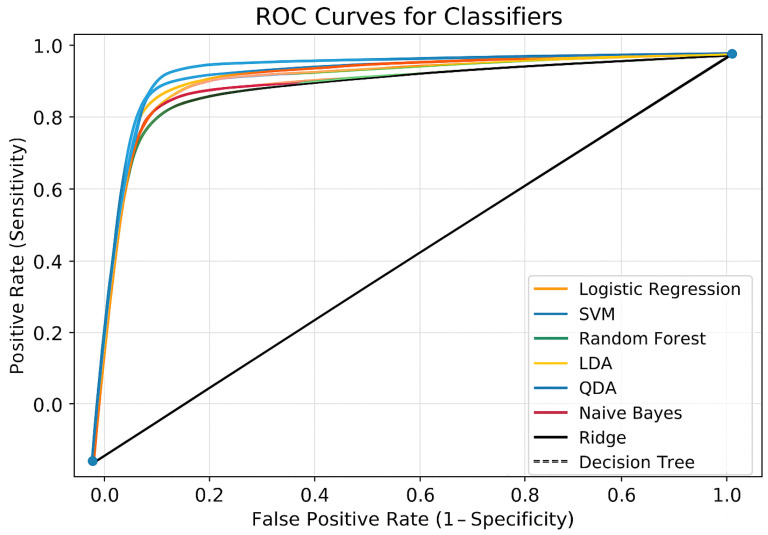
ROC curves for all classifiers.

The ROC curves are shown in Figure 1 for each classifier presented in Table 6 used in the CKD prediction. True Positive Rate (sensitivity) is represented by the *y*-axis, and False Positive Rate (1 − specificity) is represented by the *x*-axis. The random classifier’s performance is shown by the dashed diagonal line (AUC = 0.5). Every classifier showed a high degree of selective capacity by performing much better than the random baseline. These models maintain a high sensitivity (>0.85) while maintaining the false positive rate below 0.2, as seen by the close clustering of the Logistic Regression, SVM, Random Forest, Ridge, LDA, QDA, and Naïve Bayes curves. The ensemble techniques (Voting Ensemble and Stacking Ensemble) performed the best among them; their ROC curves, which are located closest to the upper-left corner, show a better balance between sensitivity and specificity. It appears that the Stacking Ensemble model performs better than any other model, indicating that merging several classifiers improves predictive power. The ROC curve of the Decision Tree classifier was marginally lower than that of the other techniques, suggesting a higher false positive rate and lower sensitivity at similar thresholds, indicating comparatively poorer performance. However, compared to random categorization, it still performs noticeably better. For CVD prediction, ensemble models, particularly the Stacking Ensemble, offer the most dependable performance, according to the ROC analysis.

**Figure 2:**
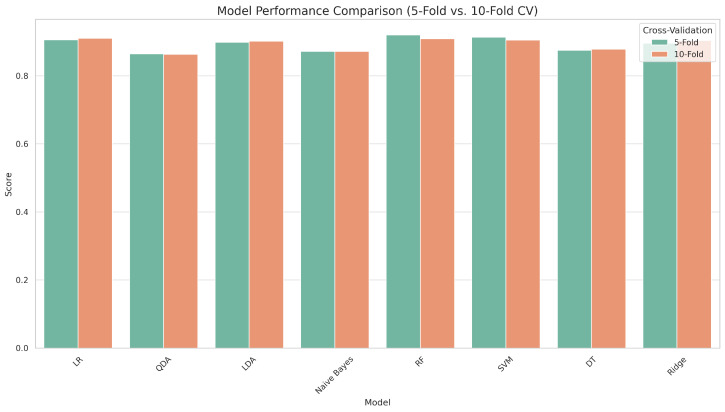
Accuracy comparison of models under 5-fold and 10-fold cross-validation.

Figure 2 compares eight machine learning models for CKD prediction using two cross-validation strategies: 5-fold and 10-fold. The models were tested using five essential metrics: accuracy, sensitivity, specificity, precision, and F1 score. RF consistently outperformed all other classifiers on most criteria, demonstrating its outstanding generalization ability and tolerance to overfitting. SVM and LR produced robust and competitive results, closely matching RF in terms of prediction accuracy. The changes in model performance between 5-fold and 10-fold cross-validation were minimal, highlighting the robustness and stability of the results across multiple validation schemes. Notably, 5-fold cross-validation achieved equivalent accuracy while requiring less computational effort, making it a more suitable option for practical applications. Models like QDA and Naïve Bayes performed worse in terms of sensitivity and specificity, indicating a limited ability to handle complex or unbalanced clinical information. Overall, the figure demonstrates the higher performance of ensemble and margin-based classifiers for CKD prediction, supporting the adoption of 5-fold cross-validation as a dependable and computationally economical model assessment method.

**Figure 3:**
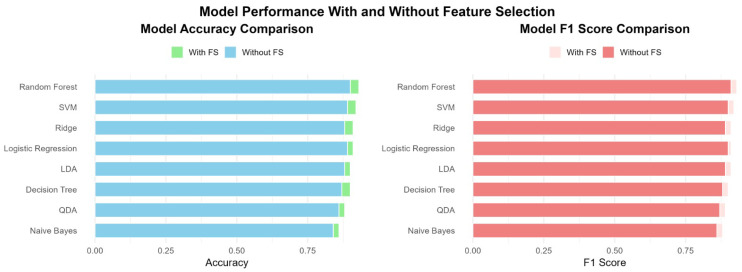
Comparative performance of machine learning models with and without feature selection based on key evaluation metrics.

Figure 3 provides a comparative visualization of model performance with and without feature selection across several evaluation metrics, including accuracy, sensitivity, specificity, precision, and F1 score. Overall, the results demonstrate that feature selection generally improves or maintains model performance. Notably, models such as QDA and SVM demonstrated significant improvements in sensitivity and F1 score after applying feature selection, indicating enhanced identification of positive cases and balanced performance. Random Forest and Logistic Regression maintained robust performance in both scenarios, highlighting their inherent strength in handling high-dimensional data. Although some models, like Decision Tree, exhibited marginal differences, feature selection led to slightly improved generalization by reducing noise and redundancy. These findings highlight the practical benefits of incorporating feature selection into machine learning pipelines, particularly in healthcare applications such as CKD prediction, where model interpretability and efficiency are crucial.

**Figure 4:**
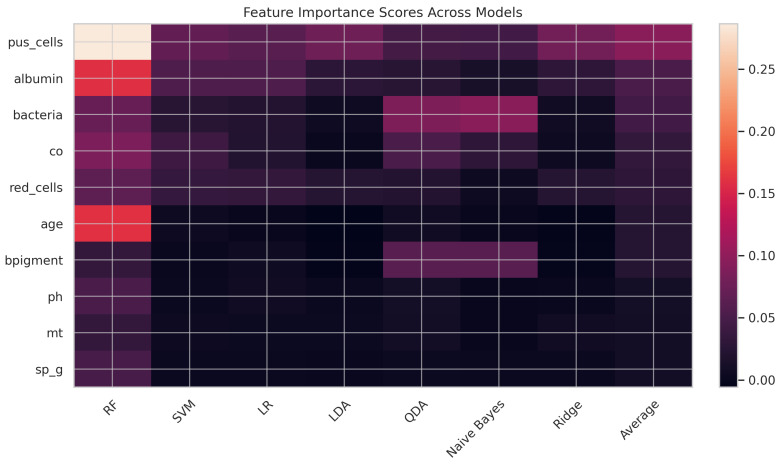
Feature importance heatmap.

The feature importance heatmap provides a comparative visualization of how various clinical features contribute to the prediction of CKD across different machine learning models, as presented in Figure 4. Each row represents a clinical feature (e.g., pus_cells, albumin, bacteria), and each column corresponds to a model (e.g., Random Forest, SVM, Logistic Regression). Warmer colors indicate higher importance scores, reflecting the feature’s relative contribution to model prediction. Among all features, pus_cells emerges as the most influential predictor, with consistently high importance across models, especially in the Random Forest (0.2866) and Logistic Regression (0.0605) models, contributing to its highest average importance score of 0.0949. Albumin and bacteria follow as the next most influential variables, with average scores of 0.0524 and 0.0452, respectively. Features like age, bpigment, and co show moderate importance, while pH, mt, and sp_g contribute minimally across all models. Thus, this visualization highlights the robustness of pus_cells and albumin as key indicators for early CKD detection, supporting their prioritization in clinical decision-making and model development. It also reveals variations in model sensitivity to specific features, guiding future refinement of feature engineering strategies.

**Figure 5:**
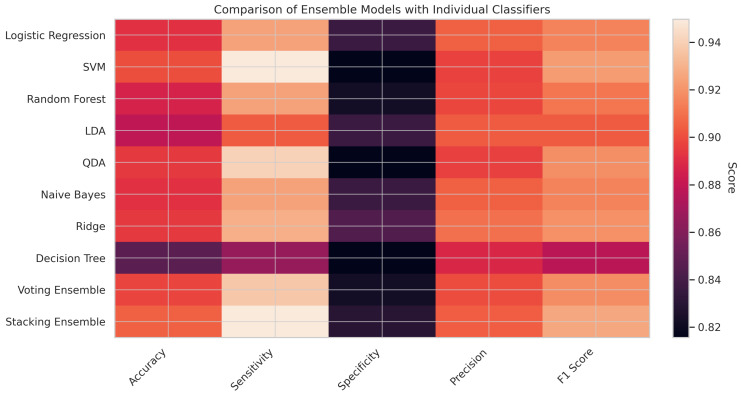
Ensemble vs. individual heatmap.

In Figure 5, presenting the heatmap comparing ensemble models with individual classifiers offers a clear and intuitive visualization of model performance across five key evaluation metrics: accuracy, sensitivity, specificity, precision, and F1 score. Among all the models, the Stacking Ensemble exhibits the highest performance, especially in accuracy (0.9053), sensitivity (0.9498), precision (0.9044), and F1 score (0.9265), indicating its superior ability to generalize and balance between false positives and false negatives. The Voting Ensemble also performs competitively, demonstrating enhanced sensitivity and an F1 score that surpasses that of most individual classifiers. In contrast, particular models, such as the Decision Tree, show comparatively lower performance across all metrics, reaffirming the value of ensemble approaches. Overall, the heatmap reinforces that Ensemble learning—particularly Stacking—can significantly enhance prediction robustness and reliability in clinical decision support systems for CKD diagnosis.

## 4. Discussion

Several ML algorithms for differentiating patients with CKD from those without the ailment are thoroughly evaluated in this study. Out of the eight classifiers that were analyzed, RF consistently produced better predictive results than the others: KNN, LR, LDA, QDA, SVM, Ridge Classifier, NB, and Regression Tree (RT). Due to its robustness and generalizability, it achieved the highest classification accuracy in both 5-fold (91.58%) and 10-fold (90.53%) cross-validation. However, the SVM model also demonstrated its effectiveness as a classifier, producing results that were consistent across validation procedures, albeit with slightly less volatility. Additionally, ensemble techniques, specifically the Voting and Stacking models, yielded encouraging results with respective accuracies of 90.53% and 89.74%. Feature selection improved model performance by reducing noise and dimensionality. The results demonstrate the feasibility of incorporating Random Forest and ensemble algorithms into decision support systems for detecting early-stage CKD using regular clinical data. The approach highlights how ML models can aid in early diagnosis, particularly in situations with limited resources or during initial clinical examinations [62,63]. These models can serve as effective supplements to established diagnostic tools, providing scalable and interpretable decision support that is particularly useful in telemedicine and rural healthcare settings. Future studies should use richer and longitudinal datasets to increase prediction power and therapeutic relevance [64,65].

On the other hand, this study has significant limitations. First, the dataset employed is cross-sectional, which limits the capacity to estimate temporal progression or early transitions between CKD stages. This shortcoming restricts its use in continuous monitoring and prognostic modeling. Furthermore, the data were obtained from a single clinical source, which may limit the model’s applicability to other geographic locations, healthcare systems, or demographic groupings. To improve the clinical usefulness and robustness of these models, future research should test them with varied, multi-center, and longitudinal data. Integrating other clinical, lifestyle, and genetic factors may further enhance the accuracy of prediction. Advanced methodologies, including deep learning architectures and more advanced ensemble tactics, should also be investigated. Furthermore, future models should prioritize interpretability and real-time application to facilitate transparent and informed clinical decision-making in real-world scenarios.

Although this work concentrates on traditional machine learning models (such as RF, SVM, and LR) due to their interpretability and simplicity, new deep learning methods have a lot of potential for CKD prediction. Advanced models such as Mamba capsule routing [66], Transformers [67], and Capsule Networks [68] have an improved capacity to simulate intricate linkages and temporal patterns, whilst CNNs and other approaches can capture spatial information. Comparison of these studies are presented in Table 8. Future research could investigate these techniques to handle richer data types, such as imaging and longitudinal recordings, and to increase diagnosis accuracy. Since the dataset was gathered from a single medical facility (BMC KPK, Pakistan), it lacked important sociodemographic characteristics (such as occupation, income, education, and ethnicity), which limited its generalizability and demographic variety. Therefore, it is advised to use caution when extrapolating the findings and to perform additional validation on bigger, multi-center, and more varied datasets.

## 5. Conclusions

This study utilizes a publicly available clinical dataset to examine machine learning algorithms for the early detection and categorization of CKD. Eight machine learning models were evaluated using the F1 score, accuracy, sensitivity, specificity, and precision metrics. Random Forest was the most successful classifier in both 5- and 10-fold cross-validation, achieving 90.53% and 91.58% accuracy, respectively. Support Vector Machines (SVMs) achieved equal accuracy rates of 91.05% and 90.00%. Ensemble techniques showed promising results, with the Voting model achieving 89.74% accuracy and the Stacking model matching the performance of Random Forest. The results underscore the importance of data-driven methods in enhancing CKD diagnosis and supporting clinical decisions, particularly in resource-constrained settings, as the model relies on a limited number of features.

However, this work demonstrates how machine learning, specifically Random Forest and Ensemble models, can effectively predict CKD early on using a limited number of clinical variables. It provides a valuable, evidence-based foundation for future decision support systems, particularly in healthcare environments with limited resources. Early detection of CKD and resource allocation can be facilitated by integrating machine learning algorithms into primary healthcare systems. In underprivileged areas, policymakers should prioritize building digital health infrastructure to provide AI-assisted diagnostic support.

## Figures and Tables

**Table 1:** Summary statistics of selected clinical variables stratified by CKD status.

Status	Variable	Mean	SD	Min	Max
0	Age	39.80	14.42	12	91
1	Age	47.69	15.96	12	90
0	Gender	1.56	0.50	1	2
0	U Clarity	1.42	0.50	1	2
0	pH	5.38	0.50	5	8
1	pH	5.68	0.57	5	8
0	Specific Gravity	1.014	0.0048	1.005	1.025
1	Specific Gravity	1.017	0.0052	1.000	1.050
0	Albumin	1.23	0.42	1	2
0	Glucose (Blood)	1.05	0.22	1	2
1	Glucose (Blood)	1.08	0.28	1	2
0	Sugar (glucosuria)	1.04	0.20	1	2
1	Sugar (glucosuria)	1.24	0.43	1	2
0	Ketones (KB)	1.01	0.12	1	2
1	Ketones (KB)	1.26	0.44	1	2
0	Bile Pigment	1.01	0.12	1	2
1	Bile Pigment	1.26	0.44	1	2
0	Urobilinogen	1.98	0.14	1	2
1	Urobilinogen	1.85	0.35	1	2
0	Blood	1.01	0.12	1	2
1	Blood	1.25	0.43	1	2
0	Pus Cells (urinary white blood cells)	1.87	0.34	1	2
0	Red Cells	1.79	0.41	1	2
1	Red Cells	1.44	0.50	1	2
0	Epithelial Cells	1.42	0.50	1	2
1	Epithelial Cells	1.70	0.46	1	2

**Table 2:** Comparison of model performance without feature selection using 5-fold and 10-fold cross-validation.

Model	Accuracy	Sensitivity	Specificity	Precision	F1 Score
5-Fold Cross-Validation					
LR	0.9026	0.9289	0.8582	0.9174	0.9231
QDA	0.8421	0.8243	0.8723	0.9163	0.8678
LDA	0.8947	0.9205	0.8511	0.9129	0.9167
Naïve Bayes	0.8474	0.8201	0.8936	0.9289	0.8711
RF	0.9158	0.9331	0.8865	0.9331	0.9331
SVM	0.9105	0.9331	0.8723	0.9253	0.9292
DT	0.8684	0.8954	0.8227	0.8954	0.8954
Ridge	0.8921	0.9205	0.8440	0.9091	0.9148
10-Fold Cross-Validation					
LR	0.9079	0.9331	0.8652	0.9215	0.9272
QDA	0.8474	0.8494	0.8440	0.9022	0.8750
LDA	0.8974	0.9205	0.8582	0.9167	0.9186
Naïve Bayes	0.8500	0.8326	0.8794	0.9213	0.8747
RF	0.9053	0.9289	0.8652	0.9212	0.9250
SVM	0.9000	0.9205	0.8652	0.9205	0.9205
DT	0.8658	0.8745	0.8511	0.9087	0.8913
Ridge	0.9000	0.9247	0.8582	0.9170	0.9208

**Table 3:** Optimal hyperparameters used for each classifier (based on grid search).

Model	Tuned Hyperparameters
Logistic Regression (LR)	penalty = l2, solver = lbfgs, C = 1.0
QDA	$reg_{param}$ = 0.01
LDA	solver = svd
Naïve Bayes	var_smoothing = 1 $\times \;10^{-9}$
Random Forest (RF)	n_estimators = 200, max_depth = 10, min_samples_split = 5
Support Vector Machine (SVM)	kernel = ‘rbf’, C = 10, gamma = 0.01
Decision Tree (DT)	max_depth = 8, min_samples_split = 5, criterion = ‘gini’
Ridge Classifier	alpha = 1.0, solver = ‘auto’

**Table 4:** Comparative analysis of model performance with and without feature selection.

Model	Accuracy	Sensitivity	Specificity	Precision	F1 Score
Without Feature Selection					
Random Forest	0.9158	0.9498	0.8652	0.9228	0.9361
SVM	0.9105	0.9414	0.8369	0.9073	0.9240
Logistic Regression	0.9026	0.9163	0.8723	0.9241	0.9202
LDA	0.8947	0.9289	0.8440	0.9098	0.9193
Ridge	0.8921	0.9289	0.8440	0.9098	0.9193
Decision Tree	0.8684	0.8703	0.8582	0.9123	0.8908
Naïve Bayes	0.8474	0.8452	0.8582	0.9099	0.8764
QDA	0.8421	0.8452	0.8369	0.8978	0.8707
With Feature Selection					
QDA	0.9079	0.9414	0.8511	0.9146	0.9278
SVM	0.9026	0.9456	0.8298	0.9040	0.9243
Random Forest	0.8974	0.9331	0.8369	0.9065	0.9196
Logistic Regression	0.8947	0.9372	0.8227	0.8996	0.9180
LDA	0.8947	0.9163	0.8582	0.9163	0.9163
Ridge	0.8947	0.9163	0.8582	0.9163	0.9163
Naïve Bayes	0.8816	0.8996	0.8511	0.9110	0.9053
Decision Tree	0.8526	0.8745	0.8156	0.8894	0.8819

**Table 5:** Feature importance scores across models.

Feature	RF	SVM	LR	LDA	QDA	Naïve Bayes	Ridge	Average
Pus Cells (urine)	0.2866	0.0687	0.0605	0.0766	0.0458	0.0453	0.0805	0.0949
Serum Albumin Level (g/dL)	0.1589	0.0553	0.0545	0.0292	0.0258	0.0132	0.0300	0.0524
Bacteria Presence (urine)	0.0717	0.0258	0.0226	0.0058	0.0884	0.0950	0.0068	0.0452
Coronary Obstruction (CO)	0.0864	0.0429	0.0213	0.0013	0.0508	0.0303	0.0045	0.0339
Red Blood Cells (urine)	0.0657	0.0355	0.0353	0.0234	0.0221	0.0047	0.0250	0.0303
Age (years)	0.1601	0.0053	0.0005	−0.0058	0.0076	0.0000	−0.0029	0.0236
Bile Pigment (urine)	0.0351	0.0018	0.0066	−0.0026	0.0616	0.0613	−0.0018	0.0231
Urine pH	0.0502	0.0026	0.0076	0.0024	0.0111	0.0005	0.0018	0.0109
Microtubule Test (mt)	0.0353	0.0053	0.0034	0.0042	0.0095	0.0005	0.0079	0.0094
Urine Specific Gravity (Sp.G)	0.0499	0.0024	0.0029	0.0011	0.0037	0.0032	0.0024	0.0094

**Table 6:** Comparison of ensemble models with individual classifiers.

Model	Accuracy	Sensitivity	Specificity	Precision	F1 Score
Logistic Regression	0.8921	0.9247	0.8369	0.9057	0.9151
SVM	0.9000	0.9498	0.8156	0.8972	0.9228
Random Forest	0.8868	0.9247	0.8227	0.8984	0.9113
LDA	0.8789	0.9038	0.8369	0.9038	0.9038
QDA	0.8947	0.9414	0.8156	0.8964	0.9184
Naïve Bayes	0.8921	0.9247	0.8369	0.9057	0.9151
Ridge	0.8947	0.9289	0.8440	0.9098	0.9193
Decision Tree	0.8474	0.8661	0.8156	0.8884	0.8771
Voting Ensemble	0.8974	0.9372	0.8227	0.8996	0.9180
Stacking Ensemble	0.9053	0.9498	0.8298	0.9044	0.9265

**Table 7:** Confusion matrix for Stacking Ensemble (*n* = 382).

Actual/Predicted	Positive	Negative	Total
Positive	228	12	240
Negative	24	118	142
Total	252	130	382

**Table 8:** Evaluation of the proposed CKD classification model in relation to previous studies.

Study	Dataset	Model/Method	No. of Features	Accuracy (%)	Remarks
Proposed Study (2025)	BMC KPK, Pakistan	RF, SVM, Ensemble (Voting, Stacking)	21	RF: 91.58 (5-fold), 90.53 (10-fold)	Feature selection improved model robustness and interpretability
Obermeyer et al. (2016) [62]	U.S. Clinical Data	Logistic Regression	14	83.4	Early application of ML in clinical risk prediction
Rajkomar et al. (2019) [63]	EHR Multi-hospital Dataset	Deep Neural Network	48	90.0	Demonstrated potential of DL in hospital mortality and disease detection
Esteva et al. (2019) [64]	Diverse Health Records	Gradient Boosted Trees	30	88.7	Highlighted interpretability vs. accuracy trade-off
Beam and Kohane (2018) [65]	U.S. Health Systems	Random Forest	25	89.1	Emphasized ML integration into clinical workflow
Zhang et al. (2025) [66]	Synthetic CKD Dataset	Mamba Capsule Routing Network	32	93.2	Showed advantages of capsule routing in complex data patterns
Xia et al. (2024) [67]	Multi-source Clinical Data	Transformer-based Classifier	40	92.6	Effectively modeled temporal dependencies in patient records
Liu et al. (2024) [68]	Hospital CKD Dataset	Capsule Network	28	91.8	Improved spatial relationship modeling over CNNs

## Data Availability

The data used in this study are available at https://www.researchgate.net/publication/372689997_Chronic_kidney_disease_patients_from_district_Buner_Khyber_Pakhtunkhwa_Pakistan (accessed on 20 January 2025).
